# Experimental data of phospholipid supplementary in full fishmeal replacement diets on growth indices, whole body nutrient composition, muscle fatty acid composition, haematocrit value and serum lysozyme activity in Malaysian mahseer (*Tor tambroides*)

**DOI:** 10.1016/j.dib.2020.105287

**Published:** 2020-02-13

**Authors:** Sohel Mian, Sairatul Dahlianis Ishak, Noordiyana Mat Noordin, Md Abdul Kader, Yazed Muhammad Abduh, Helena Khatoon, Ambok Bolong Abol-Munafi

**Affiliations:** a Department of Fish Biology and Genetics, Faculty of Fisheries, Sylhet Agricultural University, Sylhet, 3100, Bangladesh; b Institute of Tropical Aquaculture and Fisheries, Universiti Malaysia Terengganu, 21030 Kuala Nerus, Malaysia; c Faculty of Fisheries and Food Science, Universiti Malaysia Terengganu, 21030, Kuala Nerus, Malaysia; d Mainstream Aquaculture Pty Ltd, 73 Lock Ave, Werribee, VIC 3030, Australia; e Department of Aquaculture, Faculty of Fisheries, Chittagong Veterinary and Animal Sciences University, Chittagong 4225, Bangladesh

**Keywords:** Fish nutrition, Feed formulation, Nutrient supplement, Dietary phospholipid, Fatty acid, Growth performance, Haematology

## Abstract

The data collection was initiated to evaluate the effects of supplementary phospholipid to non-fishmeal based diet in order to make functional diets for the Malaysian Mahseer, *Tor tambroides*. Four iso-nitrogenous and iso-lipidic diets were formulated to consist 100% fishmeal (FM100), 0% fishmeal or full fishmeal replacement (FM0), and 0% fishmeal supplemented with 4% phospholipids (FM0+4%PL), 6% phospholipids (FM0+6%PL). A 60-day feeding trial was conducted and data collection was carried out for the following parameters; growth indices, somatic parameters, whole body nutrient composition, muscle fatty acid composition, haematocrit value and serum lysozyme activity. Fish fed FM0 diets showed significantly poor performance (P < 0.05) for all parameters. Fish showed improved growth, better whole body protein content and higher lysozyme activity when fed FM100 and phospholipid supplemented diets. In general, the current study validated that dietary PL supplementation may possibly improve the growth and survival of juvenile *T. tambroides* fed non-fishmeal based diets. Findings of this research could contribute in the aquaculture development of *T. tambroides*.

**Table:** Specifications Table

Subject	Food Science, Aquatic Science
Specific subject area	Fish nutrition, growth, haematology, serum lysozyme
Type of data	Table, Chart and Graph
How data were acquired	Physical measurements as well as calculation for growth performance and somatic indices, biochemical analyses for proximate composition, gas chromatography (GC) analysis for fatty acid profile, haematocrit value from microhaematocrit reader and serum lysozyme activity from turbidimetric assays.
Data format	Raw, analyzed and filtered
Parameters for data collection	Four experimental diets were prepared; 100% fishmeal (FM100), 0% fishmeal fully replaced fishmeal (FM0), 0% fishmeal supplemented with 4% phospholipids (FM0+4%PL), and 0% fishmeal supplemented with 6% phospholipids (FM0+6%PL). Each dietary treatment were in triplicates with 10 juvenile fish per replicate.
Description of data collection	For growth performance: final weight, weight gain, specific growth rate, feed intake, feed conversion rate, survival percentage. For somatic parameters: hepatosomatic index, viscerosomatic index, condition factor. For proximate composition: moisture, protein, lipid and ash contents on experimental diets and whole fish body. For fatty acid profile: GC analysis on experimental diets and fish muscle. For haematocrit values: percentage of erythrocytes. For serum lysozyme activity: absorbance readings from spectrophotometer.
Data source location	Institute of Tropical Aquaculture and Fisheries Research (AKUATROP), Universiti Malaysia Terengganu, 21030, Kuala Nerus, Terengganu, Malaysia and Research
Data accessibility	Data are available with this article

**Table:** 

**Value of the Data** • Phospholipid is very essential in the early stage of this fish, as they cannot be synthesized de novo. Inclusion of dietary phospholipids for Tor tambroides can reduce dependency to fish meal and can improve production of low cost feed • Addition of phospholipids as source of essential fatty acids may able to compensate the lack of essential fatty acids derived from fish meal • Effects of phospholipid supplemented diets on growth and other physiological parameters are good reference for future feeding studies on this species and other freshwater fish. • This new information is an important step in developing species-specific diets that will support expansion of this emerging aquaculture species.

## 1 Data

The formulation and proximate composition of experimental diets were shown in Table 1. The diets were iso-nitrogenous and iso-lipidic with following fishmeal and phospholipid composition; 100% fishmeal (FM100), 0% fishmeal fully replaced fishmeal with soybean meal, fish bone meal and blood meal (FM0), 0% fishmeal supplemented with 4% phospholipids (FM0+4%PL), and 0% fishmeal supplemented with 6% phospholipids (FM0+6%PL). The fatty acid compositions of each experimental diets were shown in Table 2.

**Table 1:** Feed and proximate composition of four experimental diets used in a 60-day feeding experiment of *T. tambroides* juveniles.

Ingredients/Diets (g100g^−1^)	Experimental diets			
	FM100	FM0	FM0 + 4%PL	FM0 + 6%PL
Fish Meal	45.00	0.00	0.00	0.00
Soybean meal	0.00	41.33	41.33	41.33
Fish bone meal	0.00	11.81	11.81	11.81
Blood meal	0.00	5.90	5.90	5.90
Shrimp meal^a^	2.00	2.00	2.00	2.00
Squid meal^a^	2.00	2.00	2.00	2.00
Wheat flour	25.00	19.50	19.50	19.50
Cod liver oil	2.00	4.50	4.50	4.50
Palm oil	6.00	6.00	2.00	0.00
Soybean lecithin	0.00	0.00	4.00	6.00
Vitamin Premix^b^	2.00	2.00	2.00	2.00
Mineral premix^c^	2.00	2.00	2.00	2.00
Vitamin C	0.20	0.20	0.20	0.20
CMC^d^	2.00	2.00	2.00	2.00
Alpha-cellulose	11.80	0.76	0.76	0.76
Total	100	100	100	100
**Proximate composition (g100g**^**−1**^**as fed basis)**				
Protein	46.7	46.6	47.1	46.8
Lipid	14.5	14.6	14.4	14.6
Moisture	11.2	11.4	11.3	11.5
Ash	6.8	8.8	7.0	7.5
Gross energy (kJg^−1^)^e^	19.89	18.99	19.80	19.85
Phospholipid (PL)^f^	0.35	0.22	4.22	4.24

a Raw materials purchased from local market, oven dried and made in laboratory.

b Rovithai, DSM Nutritional Products Ltd. Scotland; composition (IU g^−1^/mg kg^−1^): vitamin A 50 IU, vitamin D3 10 IU; vitamin E130 g, vitamin B1 10 g, vitamin B2 25 g, vitamin B6 16 g, vitamin B12 100 mg, biotin 500 mg, pantothenic acid 56 g, folic acid 8 g, niacin 200 g, anticake 20 g, antioxidant 0.2 g and vitamin K3 10 g.

c Rovithai, DSM Nutritional Products Ltd. Scotland; composition (g kg^−1^): copper 7.50 g, iron 125.0 g, manganese 25.0 g, zinc 125.0 g, cobalt 0.50 g, iodine 0.175 g, selenium 0.300 g and anticake 10.0 g.

d Carboxymethyl cellulose.

e Gross energy was calculated using physiological fuel values of 4.0, 4.0 and 9.0 kcal g^−1^ for protein, carbohydrate and lipid respectively [1].

f Calculated PL [2].

**Table 2:** Fatty acid composition (% of total fatty acids) of experimental diets used in a 60-day feeding experiment of *T. tambroides* juveniles.

Fatty acids/ratio	FM100	FM0	FM0 + 4%PL	FM0 + 6%PL
C4:0	9.37	–	1.08	1.40
C14:0	2.72	5.89	2.94	3.21
C14:1	–	–	1.57	0.16
C15:0	–	0.43	0.41	0.75
C15:1	0.53	2.49	4.12	5.49
C16:0	20.97	25.69	26.93	25.43
C16:1	2.72	2.96	3.33	4.07
C17:0	0.18	0.47	0.84	0.71
C17:1	0.27	0.33	0.36	0.22
C18:0	8.32	9.04	14.33	15.67
C18:1n9	16.49	25.53	17.55	14.14
C18:2n6	8.26	8.91	9.80	10.33
C18:3n6	–	0.37	1.13	1.50
C18:3n3	0.31	0.22	0.70	0.77
C20:0	0.18	0.33	0.35	0.38
C20:1	3.79	6.64	3.26	3.81
C20:3n6	–	0.11	0.60	0.85
C20:4n6	0.31	0.35	0.44	0.46
C20:3n3	2.22	1.64	–	1.59
C20:5n3	4.29	2.60	2.69	3.26
C22:0	1.73	0.23	0.45	0.54
C22:2	–	0.06	0.17	0.31
C22:6n3	10.88	4.62	0.94	0.55
C24:0	–	0.20	0.41	0.61
C24:1	0.87	0.73	6.03	3.22
∑SFA^a^	49.06	42.47	47.93	49.65
∑MUFA^b^	24.67	38.68	36.22	31.11
∑PUFA^c^	10.79	11.25	11.60	15.04
∑HUFA^d^	15.48	7.57	4.07	3.81
∑n-3^e^	17.70	9.08	4.33	6.17
∑n-6^f^	8.57	9.74	11.97	13.14
∑n-3/∑n-6	2.07	0.93	0.36	0.47

Values are means ± S.E of triplicate measurements.

Means within a row with the same superscripts are not significantly different (*P* > 0.05).

a ∑SFA, saturated fatty acid: C10:0, C12:0, C16:0, C14:0, C18:0, C17:0, C20:0, C22:0.

b ∑MUFA, monounsaturated fatty acids: C15:1, C16:1, C17:1, C18:1n-9, C20:1, C24:1.

c ∑PUFA, polyunsaturated fatty acid: C18:2n-6, C18:3n-6, C18:3n-3, C20:3n-6.

d ∑HUFA, highly unsaturated fatty acids: C20:4n-6, C20:5n-3, C22:6n-3.

e ∑n-3:18:3n-3, 20:5n-3, C22:6n-3.

f ∑n-6:18:2n-6, 18:3n-6, 20:3n-6, 20:4n-6.

The survival percentage in all dietary treatments was 100% which means that all fish were still alive at the end of the experimental period. Growth performance and feed utilization parameters of *T. tambroides* were presented in Table 3. Significant differences (P < 0.05) were observed for all parameters in fish between dietary treatments. Fish fed diet FM0 were reported to have the poorest growth performance with the lowest feed intake and the highest FCR.

**Table 3:** Growth and feed utilization parameters of *T.tambroides* juveniles fed experimental diets for 60 days.

Parameters	FM100	FM0	FM0 + 4%PL	FM0 + 6%PL
Survival (%)	100	100	100	100
FW (g)^a^	46.35 ± 0.30^c^	40.63 ± 0.66^a^	43.86 ± 0.65^b^	44.71 ± 0.65^b^^,^^c^
WG%^b^	24.49 ± 0.5^b^	13.87 ± 0.8^a^	20.21 ± 0.11^b^	21.72 ± 0.90^b^
SGR (% d^−1^)^c^	0.47 ± 0.01^c^	0.25 ± 0.01^a^	0.38 ± 0.01^b^	0.41 ± 0.02^b^^,^^c^
FI (% BW d^−1^)^d^	1.02 ± 0.01^b^	0.82 ± 0.02^a^	1.05 ± 0.01^b^	1.10 ± 0.03^b^
FCR^e^	2.20 ± 0.05^a^	3.30 ± 0.01^b^	2.80 ± 0.07^a^^,^^b^	2.70 ± 0.06^a^^,^^b^

Values are means ± S.E of triplicate measurements.

Means within a row with the same superscripts are not significantly different (*P* > 0.05).

a Final weight (g).

b Weight gain (%).

c Specific growth rate (% d^−1^).

d Feed intake (% BW d^−1^).

e Feed conversion ratio.

Somatic parameters were shown in Table 4. Differences were found in the hepatosomatic index among the treatments. Fish fed diet FM0+6%PL was observed to have the highest HSI along with PL supplemented diets while the lowest was observed in fish fed diet FM0. Higher VSI was found in the control group though not significantly different from other treatments. Good CF was observed in fish fed diet FM100 and FM0+4%PL.

**Table 4:** Somatic parameters of *T. tambroides* juveniles fed experimental diets for 60 days.

Parameters	FM100	FM0	FM0 + 4%PL	FM0 + 6%PL
HSI^a^	0.97 ± 0.01^a^	0.80 ± 0.07^a^	1.00 ± 0.10^a^	1.28 ± 0.03^b^
VSI^b^	4.04 ± 0.22^a^	3.71 ± 0.24^a^	3.77 ± 0.22^a^	3.74 ± 0.25^a^
CF^c^	1.19 ± 0.01^b^	1.14 ± 0.01^a^	1.20 ± 0.00^b^	1.16 ± 0.01^a^

Values are means ± S.E of triplicate measurements.

Means within a row with the same superscripts are not significantly different (*P* > 0.05).

a Hepatosomatic index.

b Viscerosomatic index.

c Condition factor.

Whole body proximate composition of *T. tambroides* was listed in Table 5. Protein, lipid, moisture and ash content of whole body were significantly (P < 0.05) influenced by the experimental diets. No significant difference (P > 0.05) was observed for whole body lipid content between treatments. Fish fed diet FM100 had the lowest whole body moisture content protein and the highest ash content significantly (P < 0.05). On the other hand, whole body protein was significantly lowest (P < 0.05) in fish fed diet FM0.

**Table 5:** Whole body proximate composition of *T. tambroides* fed experimental diets for 60 days.

Nutrient	Initial	FM100	FM0	FM0 + 4%PL	FM0 + 6%PL
Moisture	79.19 ± 0.08	72.75 ± 0.70^a^	76.06 ± 0.30^b^	76.71 ± 0.20^b^	76.5 ± 0.50^b^
Protein	11.51 ± 0.10	13.47 ± 0.30^b^	11.36 ± 0.20^a^	13.31 ± 0.10^b^	13.33 ± 0.20^b^
Lipid	5.15 ± 0.30	6.03 ± 0.10^a^	6.15 ± 0.07^a^	6.08 ± 0.09^a^	6.01 ± 0.80^a^
Ash	3.22 ± 0.10	3.12 ± 0.01^b^	2.95 ± 0.02^a^	3.02 ± 0.01^a^	3.00 ± 0.20^a^

Values are means ± S.E of triplicate measurements.

Means within a row with the same superscripts are not significantly different (*P* > 0.05), whereas means within a row with different superscripts are significantly different (P < 0.05).

Muscle fatty acid profile of *T. tambroides* juveniles are shown in Table 6. High levels of C18:1n9 and C20:3n6 were reported in the muscle of fish fed diet FM100. Moreover, C16:0, C18:0, C18:1n9, C18:2n6 and C22:6n3 (DHA) were the FAs contributing more in the total muscle FA. Total SFA, MUFA, PUFA and HUFA contents of muscles did not show any significant variations (P > 0.05) among the dietary treatments.

**Table 6:** Fatty acid composition (% of total fatty acids) of the muscles of *T. tambroides* juveniles fed experimental diets for 60 days.

Fatty acids/ratio		FM100	FM0	FM0 + 4%PL	FM0 + 6%PL
C10:0		–	0.18 ± 0.02	0.20 ± 0.01	0.16 ± 0.01
C14:0		2.12 ± 0.0^a^	2.16 ± 0.1^a^	2.46 ± 0.30^a^	2.53 ± 0.20^a^
C15:0		0.25 ± 0.0^a^	0.35 ± 0.02^a^	0.35 ± 0.03^a^	0.42 ± 0.00^a^
C15:1		3.68 ± 0.20^a^	3.00 ± 0.00^a^	3.16 ± 0.00^a^	3.26 ± 0.30^a^
C16:0		17.35 ± 0.70^a^	16.5 ± 0.50^a^	18.50 ± 0.60^a^	18.72 ± 0.70^a^
C16:1		3.00 ± 0.30^a^	3.00 ± 0.10^a^	3.20 ± 0.10^a^	3.23 ± 0.20^a^
C17:0		0.31 ± 0.00^a^	0.27 ± 0.01^a^	0.35 ± 0.00^a^	0.24 ± 0.00^a^
C17:1		0.33 ± 0.01^a^	0.30 ± 0.03^a^	0.25 ± 0.01^a^	0.16 ± 0.00^a^
C18:0		12.96 ± 0.50^a^	12.65 ± 0.30^a^	14.10 ± 0.60^a^	13.98 ± 0.70^a^
C18:1n9		20.56 ± 0.80^b^	15.75 ± 0.90^a^	15.85 ± 0.33^a^	15.97 ± 0.40^a^
C18:2n6		9.50 ± 0.30^a^	9.51 ± 0.40^a^	10.34 ± 0.60^a^	11.67 ± 0.90^b^
C18:3n6		–	0.12 ± 0.00	0.19 ± 0.01	0.63 ± 0.03
C18:3n3		0.55 ± 0.01	0.50 ± 0.02	0.56 ± 0.01	–
C20:0		0.12 ± 0.00^a^	0.14 ± 0.01^a^	0.14 ± 0.01^a^	0.14 ± 0.01^a^
C20:1		3.52 ± 0.30^a^	4.01 ± 0.20^a^	4.10 ± 0.10^a^	4.30 ± 0.10^a^
C20:2		0.44 ± 0.01	0.50 ± 0.03	–	0.49 ± 0.00
C20:3n6		0.44 ± 0.02^b^	0.25 ± 0.00^a^	0.30 ± 0.05^a^	0.37 ± 0.02^a^
C20:4n6		1.20 ± 0.10^b^	1.70 ± 0.03^b^	0.85 ± 0.01^a^	1.16 ± 0.00^b^
C20:5n3		1.75 ± 0.20^a^	2.25 ± 0.10^b^	1.80 ± 0.00^a^	2.11 ± 0.30^b^
C22:6n3		12.67 ± 0.60^a^	11.30 ± 0.70^a^	14.50 ± 0.50^a^	12.76 ± 0.45^a^
C24:1		5.61 ± 0.02^a^	7.33 ± 0.10^b^	7.90 ± 0.30^b^	7.58 ± 0.60^b^
∑SFA^a^		33.11 ± 0.76^a^	32.36 ± 0.61^a^	36.10 ± 0.90^a^	36.27 ± 0.83^a^
∑MUFA^b^		36.70 ± 0.57^a^	33.39 ± 0.30^a^	34.46 ± 0.20^a^	34.50 ± 0.09^a^
∑PUFA^c^		10.49 ± 0.30^a^	10.38 ± 0.20^a^	11.39 ± 0.19^a^	12.67 ± 0.11^a^
∑HUFA^d^		15.62 ± 0.40^a^	15.25 ± 0.35^a^	17.15 ± 0.40^a^	16.03 ± 0.35^a^
∑n-3^e^		14.97 ± 0.07^a^	14.05 ± 0.03^a^	16.86 ± 0.12^b^	14.87 ± 0.15^a^
∑n-6^f^		11.14 ± 0.09^a^	11.58 ± 0.03^a^	11.68 ± 0.07^a^	13.83 ± 0.10^b^
∑n-3/∑n-6		1.34 ± 0.04^b^	1.21 ± 0.00^a^	1.44 ± 0.05^b^	1.08 ± 0.02^a^

Values are means ± S.E of triplicate measurements.

Means within a row with the same superscripts are not significantly different (*P* > 0.05).

a ∑SFA, saturated fatty acid: C10:0, C12:0, C16:0, C14:0, C18:0, C17:0, C20:0, C22:0.

b ∑MUFA, monounsaturated fatty acids: C15:1, C16:1, C17:1, C18:1n-9, C20:1, C24:1.

c ∑PUFA, polyunsaturated fatty acid: C18:2n-6, C18:3n-6, C18:3n-3, C20:3n-6.

d ∑HUFA, highly unsaturated fatty acids: C20:4n-6, C20:5n-3, C22:6n-3.

e ∑n-3:18:3n-3, 20:5n-3, C22:6n-3.

f ∑n-6:18:2n-6, 18:3n-6, 20:3n-6, 20:4n-6.

**Fig. 1:**
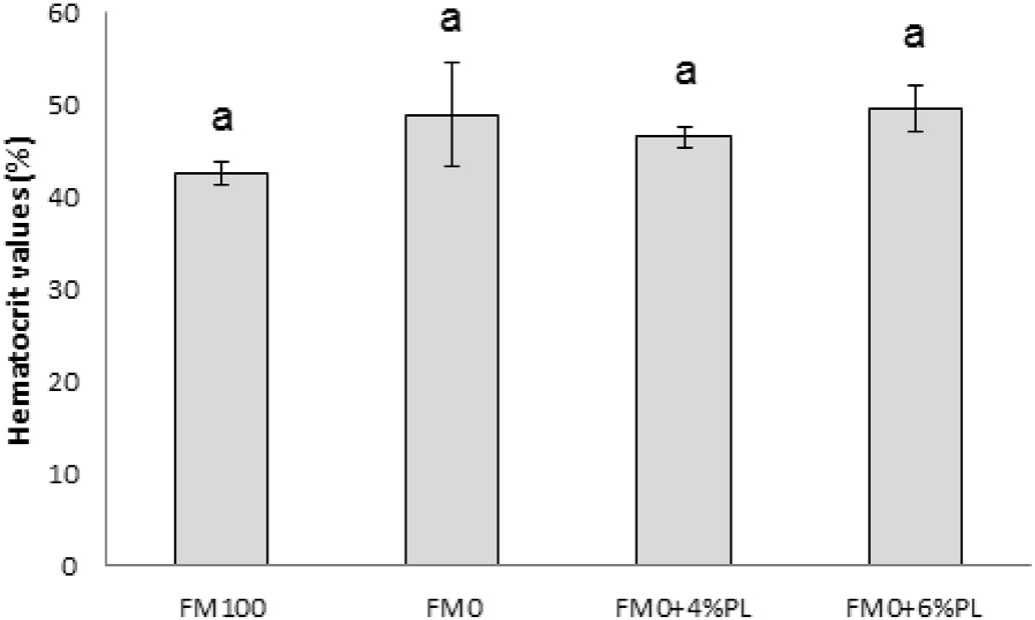
Hematocrit values of *T. tambroides* juveniles fed experimental diets for 60 days.

The hematocrit values were not significantly different among all the treatments after 60 days feeding trial as shown in Fig. 1.

**Fig. 2:**
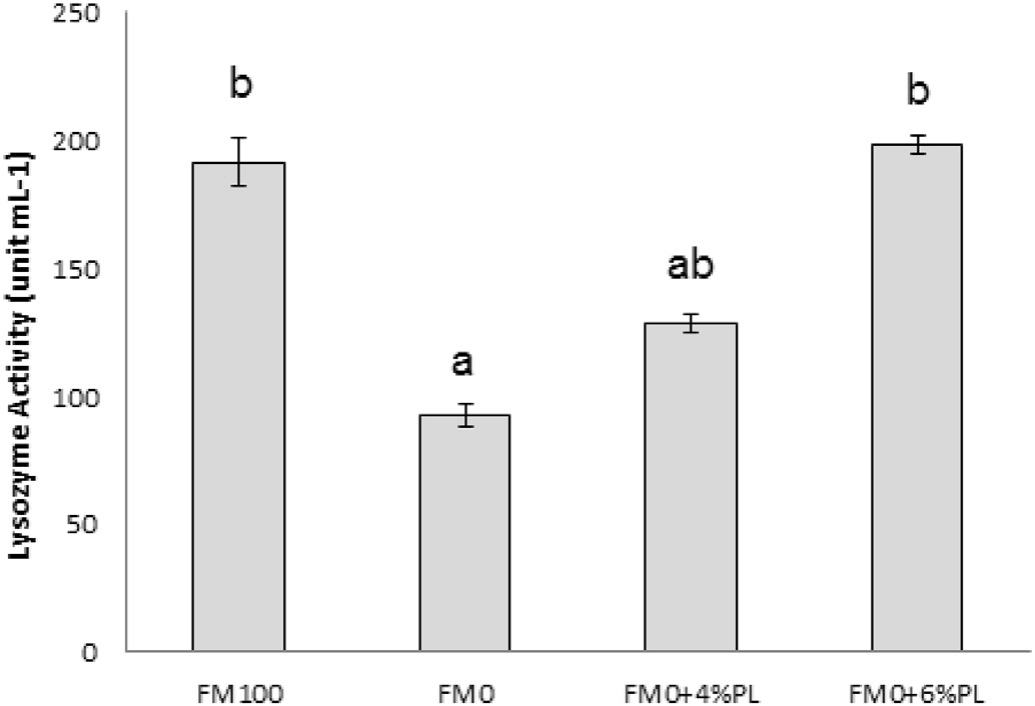
Lysozyme activity (unit mL^−1^) of blood serum of *T. tambroides* fed experimental diets for 60 days.

No significant difference (P > 0.05) was observed in serum lysozyme activity (LA) between control and PL supplemented fish groups as shown in Fig. 2. However, LA was significantly lowest between treatments.

## 2 Experimental design, materials, and methods

### 2.1 Feed formulation and preparation of experimental diets

All dietary ingredients were obtained from commercial sources. Four iso-nitrogenous and iso-lipidic diets were formulated with the following fishmeal and phospholipid composition; 100% fishmeal (FM100), 0% fishmeal fully replaced fishmeal with soybean meal, fish bone meal and blood meal (FM0), 0% fishmeal supplemented with 4% phospholipids (FM0+4%PL), and 0% fishmeal supplemented with 6% phospholipids (FM0+6%PL). All ingredients were ground through a sieve (500-μm mesh) and then thoroughly blended with the lipid components and water using a kitchen mixer. The mixtures were pelleted with a pelletizer and oven-dried for 8 h at 65 °C. Diets were then stored in plastic containers and kept dry at −20 °C until further use.

### 2.2 Fish maintenance and experimental design

Juvenile *T. tambroides* were purchased from commercial fish traders and reared in the Freshwater Hatchery, Faculty of Fisheries and Food Science, Universiti Malaysia Terengganu, Malaysia. Prior to the initial stocking, the fish were acclimatized for 10 days in the laboratory condition by maintained with commercial diet (Cargill Malaysia Sdn Bhd, Malaysia).

After acclimatization, twelve 150 L fibre glass tanks with closed water system connected to a biological filtration system were randomly stocked with 10 juveniles (initial weight; 35.0 ± 0.6g) each. All tanks were covered with fine wire to prevent fish escapes from the culture system and maintained a constant flow of aeration under natural light and dark regime. The fish were hand fed with the respective test diets at visually near satiation, twice daily for 60 days. Experimental fish were weighted in bulk at every two weeks interval to determine growth and health condition. The water quality parameters were monitored daily and data showed that temperature, pH and DO varied between 28.7 and 30.1 °C, 6.9 to 9.2 and 5.5–7.5 mg l^−1^, respectively.

### 2.3 Feeding trial

At the beginning of the trial, 10 fish were sampled and kept at −20 °C for further proximate composition analysis. At completion of feeding trial, fish were maintained without feeding for 24 hours, and then anaesthetized using clove oil (50 μl/l) before final sampling. The total number, length, and weight of each fish was taken from every single replicated tank and recorded accordingly. Four fish from each replicate tank were sampled and preserved at −20 °C for final whole body proximate composition. Intestine and liver were collected from three fish of each replicate tank and weighed to calculate viscerasomatic index and hepatosomatic indices. Liver samples were pooled together and stored at −80 °C before further analysis. All experimental feeding and fish sampling methods were done in accordance to the animal ethical guidelines by Universiti Malaysia Terengganu, Malaysia.

### 2.4 Data collection and calculation of growth indices

Growth indices of the fish were appraised in terms of final weight, weight gain, and specific growth rate. Feed utilization parameters such as feed intake, feed conversion ratio and condition factor were estimated as well.

At the end of each trial, data of the growth performance and physiological status of experimental fish were evaluated and constructed on the following formulas:

$$\text{WG, \%}=\frac{\left(\text{final body weight}-\text{initial body weight}\right)\text{x }100}{\text{initial body weight}}$$

$$\text{SGR, \%}=\left[\frac{\text{ln final body weight}-\text{ln initial body weight}}{\text{experimental days}}\right]\text{x }100$$

FCR=total dry feed consumed (g)/Wet Weight Gain (g)

FI = (total dry feed given – total dry remaining diet recovered)/no. of fish.

Physiological distinctions like hepato-somatic index (HSI), viscera-somatic index (VSI) and condition factor were also assessed. Six fish per treatment were individually weighed, sacrificed and dissected. Their liver and viscera were then extracted and weighed to determine the HSI and VSI using the following formulae:

$$\text{HSI, \%}=\frac{100\text{ × liver weight (g)}}{\text{body weight (g)}}$$

$$\text{VSI, \%}=\frac{100\text{ × visceral weight (g)}}{\text{body weight (g)}}$$

Survival, % = 100 × (final no. of fish/initial no. of fish).

Condition factor CF, % = weight of fish/(length of fish)^3^ × 100.

### 2.5 Proximate composition

The proximate composition of experimental diets and fish whole body samples were analyzed using standard methods by AOAC [3]. The samples were dried to constant weight at 105 °C to determine moisture content. Crude protein content was determined by measuring nitrogen (N × 6.25) using the Kjeldahl method (2300-Auto-analyzer, FOSS, Denmark), crude lipid content by ether extraction using Soxhlet method (36680-analyer, BUCHI, Switzerland), and ash content by combustion at 550 °C for 12 h.

### 2.6 Lipid extraction and fatty acid analysis

Total lipid was extracted from samples by homogenization in chloroform/methanol (2:1, v/v) methylated and transesterified into fatty acid methyl esthers (FAME) with boron based using a one-step method with few modifications [4]. Nanodecaenoic acid, C19:0 (Fluka) with a final concentration of 1 mg mL^−1^ in hexane was used as the internal standard. Samples (200mg) were mixed with 4 mL of hexane and 1 ml of internal standard solution in a 50 mL centrifuge tube before adding 2 mL of 14% BF3 in methanol. The tubes were flushed with nitrogen gas and then closed tightly with Teflon-lined screw-caps before heating in a water bath at 100 °C for 120 min with continuous stirring. After cooling to room temperature, 1 mL of hexane was added followed by 2 mL of distilled water. The tube was then shaken vigorously for 1 min and centrifuged for 3 min at 2500 rpm. The upper phase was the hexane layer containing FAME. Finally, 2 mL of the hexane layer was transferred using a Pasteur pipette into a clean sample vial to be injected into the GC for FAME analysis. FAME samples were analyzed in an Agilent 7890 N gas chromatograph (Agilent Technologies, Inc., USA) equipped with a split injector, a flame ionization detector and a Supelco SP-2330 capillary column (30  m × 0.25 mm ID, 0.20 μm film thickness) (Supelco Inc., USA). Sample was injected to the gas chromatography by an automatic sampler unit. Nitrogen was used as the carrier gas at a rate of 40 mL min^−1^. Column temperature was set at 100 °C for the first 2 min, and then increased to 170 °C at 10 °C min^−1^ with a holding time of 2 min, followed by an increase to 200 °C at 7.5 °C min^−1^ with a holding time of 20 min. Injector and detector temperature were set at 250 and 300 °C, respectively. Individual fatty acids were identified by comparing relative FAME peak retention time of samples with those of known standards (Menhaden oil and Supelco 37 Component FAME Mix, Supelco Inc., USA). The quantification of FAME was determined as the percentage of area under chromatographic peaks over the total area of peaks.

### 2.7 Blood collection and haematological parameters

Blood was withdrawn by puncturing the caudal vein of individual fish using 1ml tuberculin syringe then kept in 2 ml micro tubes. Heparinized syringe was used for whole blood collection to analyze haematological parameters and non-heparinized syringe was used for serum. Serum samples were separated by centrifugation at 3000×*g* for 15 min at 4 °C and preserved at −80 °C for further investigation. Heparinized whole blood was used to determine haematocrit values using microhaematocrit reader and the values were expressed in percentage of erythrocytes.

### 2.8 Serum lysozyme activity

Serum lysozyme activity (LA) was determined following turbidimetric assays [5]. Serum samples of about 10 μL was added in well of microplate. Substrate samples of 190 μL/ml (0.2 mg *Micrococcus lysodeikticus*, lyophilized cell, Sigma, USA) phosphate buffer solution, pH 7.4. Absorbance readings were quantified after 1 and 5 minutes, incubation with gentle shaking at constant room temperature at 450 nm with Immuno MiniNJ-2300 spectrophotometer (System instructions, Tokyo, Japan). A unit of enzyme activity was defined as the amount of enzyme that causes a decrease in absorbance of 0.001/min.

### 2.9 Statistical analysis

All data were analyzed using One-way ANOVA and Duncan's multiple range tests with percentage values arcsine transformed before subjected to statistical analysis. Statistical analyses were performed in SPSS 21.0 for Windows (SPSS Inc., Chicago, IL, USA) and significance level of 5% (*P* < 0.05) was used for all comparisons.
